# Supplementary material for: Characterization of Differentially Expressed Circulating miRNAs in Metabolically Healthy versus Unhealthy Obesity
Source: Biomedicines. 2021 Mar 21;9(3):321. doi: 10.3390/biomedicines9030321 (PMC8004231; doi:10.3390/biomedicines9030321)
Supplement: Supplementary file 1 [file biomedicines-09-00321-s001.zip › Supplementary table 2.docx]

Supplementary table 2. Experimentally validated targets of the 3 selected miRNAs.

| **miRNA** | **Targets** | **PMID** |
| --- | --- | --- |
| hsa-miR-588 | GRN | 27571908 |
|  | DCAF7, CHAF1B | 23446348 20371350 |
|  | LHFPL2, BTG2 | 20371350  21572407 |
|  | TMEM154, SATB1, DNASE2, ZNF584, GMPS | 21572407  23446348 |
|  | ZNF665, THAP1, TXNL1 | 23446348 22012620 |
|  | ATCAY, DBT, SLC4A1 | 23446348 23824327 |
|  | TM4SF5 | 21572407  22012620 |
|  | WDR91, ZNF101, GPR156, IFNAR1, MIOX | 23313552  23824327 |
| hsa-miR-6796-5p | SHOC2, ZNF703 | 23446348  21572407 |
|  | TXLNA | 21572407  23592263 |
|  | C5ORF51 | 23313552  21572407 |
|  | ASCL2 | 23592263  24398324 |
|  | NACC2, HNRNPA0 | 23446348  20371350 |
|  | VMA21 | 20371350  21572407 |
|  | SYNGAP1 | 23824327  19536157 |
| hsa-miR-4697-3p | RRAS2 | 23592263  22012620 |
|  | SLC7A5 | 24398324  23592263 |
|  | C9orf40 | 21572407  23446348 |
|  | CHAC1 | 20371350  21572407 |
|  | SPPL2A | 24398324  21572407 |
|  | ZBTB18, LNPEP | 20371350  23446348 |

Source: miRCarta v1.1 (https://mircarta.cs.uni-saarland.de)
